# Motivation to work and attitudes towards retirement among physicians

**DOI:** 10.1186/s12913-024-11296-2

**Published:** 2024-07-25

**Authors:** Franziska U. Jung, Erik Bodendieck, Melanie Luppa, Steffi G. Riedel-Heller

**Affiliations:** 1 Institute of Social Medicine, Occupational Health and Public Health, Medical Faculty, University of Leipzig, Ph.-Rosenthal-Str. 55, 04103 Leipzig, Germany; 2 General Practice, Dresdner Straße 34a, 04808 Wurzen, Germany

**Keywords:** Retirement, Workload, Burnout, Job satisfaction, Physician

## Abstract

**Background:**

The healthcare system is currently in a state of tension due to a shortage of physicians, the early retirement of health care professionals and an increasing need for care within an (aging) society. Therefore, the aim of the present study was to examine physicians’ attitudes towards retirement and possible influencing factors on their motivation to work.

**Method:**

Data were collected as part of a baseline survey of a long-term study. The sample includes a variety of physicians (*n* = 625), working in outpatient or inpatient care, who have not yet reached the retirement age of 67. The primary outcome was to survey attitudes towards retirement using the Motivation to Work scale. Work-related characteristics (e.g., with regard to contract or working hour) as well as job satisfaction, overall health, and burnout were also included in the analyses (correlations and linear regression models).

**Results:**

According to the results, sociodemographic characteristics are not significantly related to motivation to work, whereas the other parameters (satisfaction, health, and burnout) influence attitudes towards retirement significantly.

**Conclusions:**

The results underline the need to improve the occupational conditions of physicians across different medical settings. More research is needed to understand physicians‘ decision-making with regard to retirement, especially in terms of work-related characteristics and differences.

**Supplementary Information:**

The online version contains supplementary material available at 10.1186/s12913-024-11296-2.

## Introduction

The healthcare system is currently facing a number of challenges, including a shortage of staff in all medical disciplines. At the same time, demands for healthcare services are increasing, a problem that affects many European countries and is also addressed in the context of legal regulations, as many countries aim to retain retiring physicians [1]. In Germany, for example, the statutory age limit of 68 for physicians was abolished on January 1, 2009, allowing doctors to be available for the healthcare system for longer. Especially during the COVID-19 pandemic, efforts to keep physicians working have been enforced in order to respond to rising needs in patient care [2] or respond to increases in workload [2]. In general, understanding the role of motivation to work and decisions regarding retirement timing is important in times of rising needs within the healthcare sector.

According to a systematic review [3], there is inconsistent evidence regarding physicians’ expected and actual retirement age (usually between 60 and 69 years of age). With regard to timing, some studies indicate that a large part of the medical profession, for instance, working in general medicine or emergency departments, would like to retire early [4–6]. Whereas others find a growing number of physicians that stay in the profession despite having reached retirement age [7, 8]. The decision to retire is highly complex and affected by personal (such as workplace frustration, workload pressure, job dissatisfaction, or finances), health, family, and societal factors. In the past, burnout (especially emotional exhaustion) has been suggested as a risk factor for early (involuntary) retirement and wishes to leave clinical practice among different medical specialties [9–11].

Physicians’ early retirement is of great relevance, as a shortage of physicians may hinder optimal patient care [12]. So far, study results with regard to physicians are still inconsistent and do not cover all aspects relevant in order to understand the association between personal as well as work-related factors and attitudes towards retirement [3]. In this context, entering a work arrangement as well as the duration of participation (until retirement) depend on motivation to work [13, 14]. Indeed, despite being described as highly relevant by retirement researchers, work-related motivations are poorly understood [13, 14]. Therefore, the overall aim of this study was to build up on recent literature and gain more evidence by investigating motivation to work in the context of retirement attitudes in a broad sample of physicians. Secondary, we aimed to analyze the influence of sociodemographic, health, and work-related factors on these attitudes. According to the aforementioned literature, we hypothesize that burnout is positively associated with attitudes towards retirement, as greater symptoms of burnout may increase the motivation to retire. On the other hand, we assume that overall health status and job satisfaction may have a positive effect. In other words, better health and greater job satisfaction may decrease the motivation to retire. The influence of sociodemographic factors and work-related characteristics will be investigated exploratively using a broad sample of physicians working in the eastern part of Germany. Investigating motivation to work and attitudes towards retirement in physicians is important to understand the process of work-retirement transition and may help policymakers design and implement strategies to keep physicians longer within patient care.

## Method

### Study population

The data sample is based on a baseline survey investigating the subject of working hours and retirement. Originally, 2,997 physicians working in the Federal State of Saxony were contacted by mail and asked to fill out a questionnaire anonymously. The physicians were randomly selected based on methodological considerations. Nine age cohorts were formed: the first cohort includes physicians aged 26 to 30, the second from 31 to 35, and so on. In order to achieve a sufficient cohort size for statistical analyses, and to collect enough participants for the follow-up examinations, 100 people per cohort were aimed for. Based on a usual response rate of 30%, around 333 physicians per cohort were contacted, resulting in a total of 2997 across all cohorts. In the current sampling, the overall response rate was 32% (*n* = 1,001). In the current secondary data analysis, a sub-sample was selected, which includes physicians who do not practice family medicine and have not yet exceeded the statutory retirement age in Germany (67 years of age). A wider range of physicians (for example internists or anesthesiologists) with different specialties were included to ensure that the topic under investigation is fully covered. The sample includes employed and self-employed physicians, working in outpatient and inpatient care with direct patient contact (*n* = 625). The study was approved by the ethical committee (Medical Faculty, University of Leipzig, reference number: 478/19-ek). Participants have given consent for their data to be used.

Questionnaire design and content.

Besides sociodemographic variables (age, gender, marital status, income) work-related variables were also surveyed. In this context, we included questions working time (whether they work full-time or part-time), medical setting (whether they work in inpatient or outpatient care), and working condition (permanent contract, fixed-term contract, self-employed).

The primary outcome of the present study (attitudes towards retirement) was measured using the *Motivation to Work Scale*. This instrument was developed as part of the German LidA cohort study of work, age, health, and work participation (www.lida-studie.de [13, 15]. The scale consists of five items, that focus on attitudes towards retirement (please see Appendix). A mean score is calculated ranging from 1 (low motivation to work) and 5 (high motivation to work) (see Table 1). The Cronbach alpha of this scale was 0.81. Attitudes towards retirement were measured by this scale, because “[…] retirement is considered a process reflecting decades of work exposure affecting an individuals’ MTW^1^ and their pathway towards (quality and timing of) retirement.“ (p. 1643, [14]). Therefore, by asking about the motivation to work, attitudes towards retirement can be derived based on the answers, as has been described previously [13, 15].

**Table 1:** Descriptive information on study variables

	Overall sample (*n* = 625)	Motivation to work^3^ ^Mean (SD)^
**Age in years** (*n* = 625)	44.0 (SD: 11.5)	
25–29 30–39 40–49 50–59 60–66	53 (9.8%) 124 (22.8%) 224 (41.2%) 58 (10.7%) 84 (15.5%)	2.9 (SD: 0.9) 3.1 (SD: 0.9) 3.0 (SD: 0.9) 2.7 (SD: 0.9) 2.9 (SD: 1.0)
**Gender** (*n* = 625)		
Male Female	254 (40.6%) 371 (59.4%)	3.0 (SD: 0.9) 2.9 (SD: 0.9)
**Marital status** (*n* = 625)		
Single In a relationship/married	112 (17.9%) 513 (82.1%)	3.0 (SD: 1.0) 2.9 (SD: 0.9)
**Income**^1^(*n* = 606)		
< 4.000 4.001-8.000 8.001-12.000 > 12.001	142 (22.7%) 321 (51.4%) 115 (18.4%) 47 (7.5%)	2.9 (SD: 0.9) 3.0 (SD: 0.9) 2.9 (SD: 1.0) 3.2 (SD: 0.9)
**Working hour arrangement**^2^(*n* = 519)	37.5 (SD: 7.0)	
part-time Full-time	149 (28.7%) 370 (71.3%)	2.8 (SD: 0.8) 3.0 (SD: 1.0)
**Medical Setting** (*n* = 623)		
Outpatient Inpatient	463 (74.3%) 160 (25.7%)	2.9 (SD: 0.9) 3.0 (SD: 1.0)
**Contract** (*n* = 620)		
Permanent contract Temporary contract Self-employed	331 (53.4%) 193 (31.1%) 96 (15.5%)	2.9 (SD: 0.9) 3.1 (SD: 0.9) 3.0 (SD: 1.0)
**Burnout (CBI)** ^**4**^		
Personal (*n* = 620) Work-related (*n* = 619) patient-related (*n* = 614)	45.4 (SD: 18.8) 35.6 (SD: 17.9) 22.4 (SD: 18.0)	
**Job satisfaction** ^**5**^ (*n* = 599)	18.4 (SD: 2.1)	
**Overall health status** ^**6**^ (*n* = 624)	80.7 (SD: 14.9)	

*Note*^1^= monthly household net income in Euros; ^2^= does not include self-employed physicians; part-time meaning less than 40 h/week; ^3^= range: 1–5, higher value indicating higher motivation to continue working; ^4^= Copenhagen Burnout Inventory, higher value indicating more burnout, range: 0-100 ; ^5^= higher value indicating being more satisfied, range: 11–20; ^6^= higher value indicating better health, range: 8-100

Burnout was determined using the Copenhagen Burnout Inventory (CBI) which has been adapted in order to be used for employees working in health care [15–17]. The CBI includes 19 items on three dimensions that capture different aspects of burnout (personal burnout, patient-related burnout, and work-related burnout) on a five-point scale (please see Appendix). Example items include the following: “How often are you physically exhausted?” (100 = always/ 0 = never, sub-scale *personal burnout*: Cronbach alpha = 0.89), “Do you feel burned out because of your work? (100 = to a very high degree, 0 = to a very low degree, sub-scale *work-related burnout*: Cronbach alpha = 0.87) and “Do you find it hard to work with patients?” (100 = to a very high degree, 0 = to a very low degree, sub-scale *patient-related burnout*: Cronbach alpha = 0.86).

Job satisfaction was assessed using the Weyer and colleagues scale [18]. The scale consists of eight questions and participants can either “agree” or “disagree” with each statement (please see Appendix). The higher the value of the resulting total score, the greater the job satisfaction. The Cronbach alpha of the job satisfaction scale was 0.79.

The overall state of health of the respondents was surveyed using the visual analogue scale [19, 20]. The response scale ranges from 0 (worst health) to 100 (best health, please see Appendix).

### Data analysis

In a first step, the data analysis was carried out descriptively and by means of correlation analyses. In this context, Pearson r was used for continuous variables and Spearman r for categorical variables. In a further step, multivariate linear regression models were calculated in order to examine the influence of burnout, health and job satisfaction on motivation to work. Neither multi-collinearity nor heteroscedasticity were detected within the data. A significance level of 0.05 was assumed in all analyses. The sum score of the Motivation to Work scale was used as the outcome variable. The following variables were used as independent variables: personal burnout (Model 1), patient-related burnout (Model 2), work-related burnout (Model 3), job satisfaction (Model 4), and overall health status (Model 5). The following variables were included as covariates across all models: age, gender, marital status, and income. Statistical evaluation was carried out using the statistical software program Stata SE 16.0.

## Results

Regarding sociodemographic characteristics and working conditions, descriptive information can be summarized as follows. Participants in this sample were on average 44 years old. The majority was female (59.4%), in a relationship or married (82.1%), working full-time (71.3%) and in an outpatient setting (74.3%). More than half of the physicians in this sample had a permanent contract (53.4%) and 15.5% was self-employed (Table 2).

**Table 2:** Correlation analysis between variables under investigation (r and significance)

Variable	1.	2.	3.	4.	5.	6.	7.	8.	9.	10.	11.	12.
1. Age^2^												
2. Gender^1^	-0.11**											
3. Marital status^1^	-0.02	0.12**										
4. Income^1^	0.32***	-0.20***	-0.30***									
5. Working time^1^	-0.11*	-0.26***	0.07	0.06								
6. Medical Setting^1^	0.37***	0.05	0.002	0.10*	-0.14**							
7. Working condition^1^	-0.16***	0.01	0.03	-0.12**	0.03	0.39***						
8. Personal B^2^	-0.18***	0.15**	0.04	-0.16***	-0.01	-0.10*	0.04					
9. patient-rel. B^2^	-0.08*	-0.04	0.02	-0.03	0.04	0.13**	-0.14***	0.49***				
10. work-related B^2^	-0.13**	0.11**	0.05	-0.16***	0.02	-0.06	-0.08*	0.83***	0.60***			
11. Job satisfaction^2^	0.20***	-0.06	-0.05	0.19***	-0.07	0.09*	-0.06	-0.49***	-0.46***	-0.58***		
12. overall health^2^	-0.11**	-0.05	-0.02	0.14***	0.04	-0.06	0.02	-0.47***	-0.23***	-0.46***	0.23***	
13. attitudes towards retirement^2^	-0.05	-0.07	0.02	0.03	0.10*	0.08*	0.09*	-0.29***	-0.21***	-0.31***	0.32***	0.25***

*Note*^1^=categorical variable; ^2^=continuous variable; Pearson r for continuous and Spearman r for categorical variables; B = burnout * *p* < 0.05, ** *p* < 0.01, *** *p* < 0.001

**Fig. 1:**
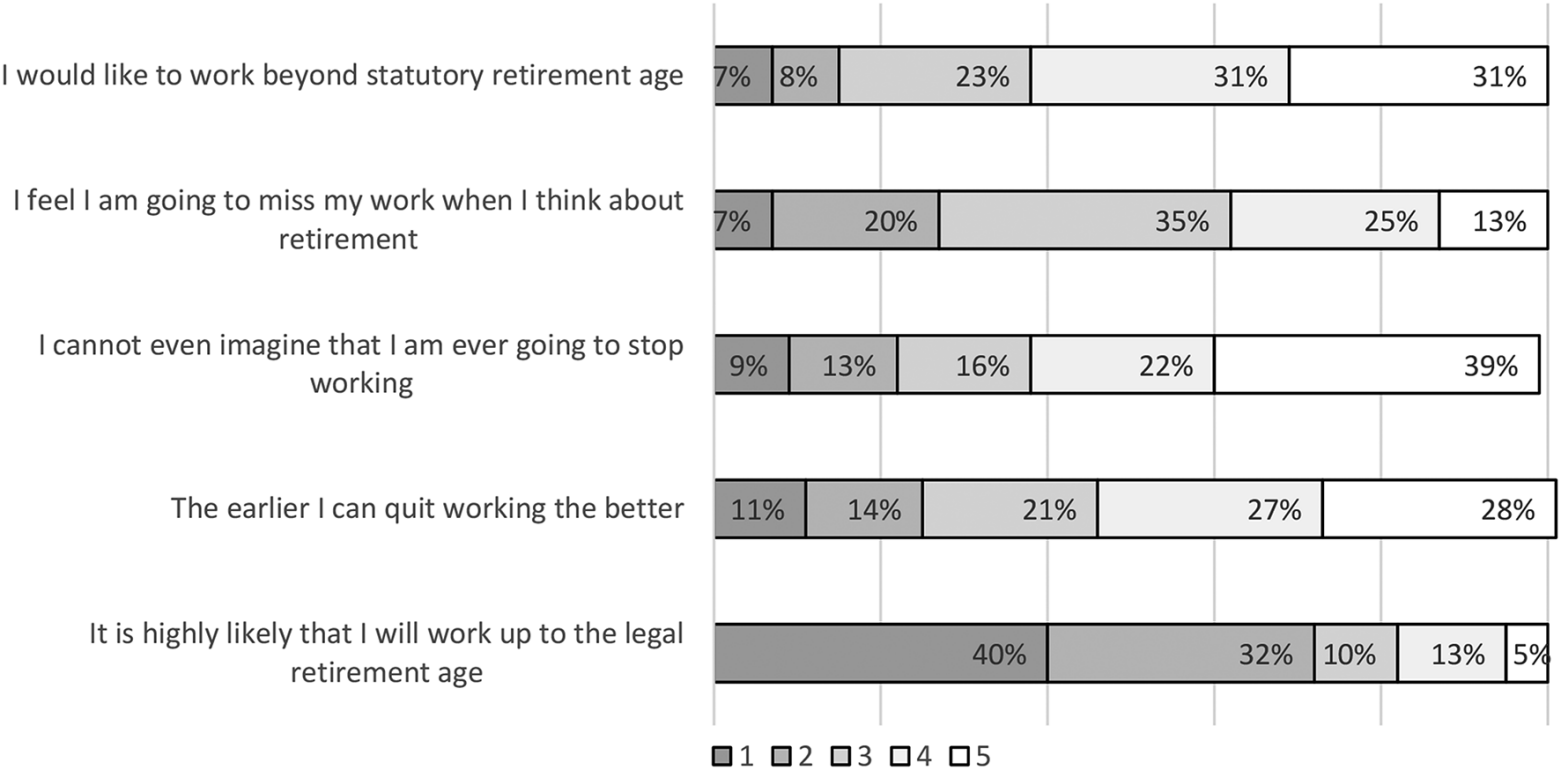
Mean scores for retirement attitudes and motivation to work. *Note* 1- fully applies; 5 – does not apply at all

Regarding retirement attitudes and motivation to continue working, the following results were obtained. Overall, 40% of physicians wanted to work up to retirement age, compared to 5% who stated that they will not work up retirement age. Moreover, „The earlier I can quit working the better“ did not apply to about 28% of physicians in this sample, whereas 11% of physicians completely agreed and 14% partly agreed with this statement. Only 7% of participating physicians stated that they are likely to work beyond retirement age, whereas 31% of physicians did not agree to this statement (Fig. 1).

Results of correlation analyses (Table 1) indicate that working full-time, working in outpatient care, being self-employed or having a temporary contract, greater subjective health, and greater job satisfaction were associated with greater motivation to work (instead of retiring). Higher burnout on all three dimensions was associated with more positive attitudes towards retirement. In terms of motivation to work and attitudes towards retirement, no significant associations were found with regard to age, gender, marital status or income.

In order to further investigate the association between motivation to work and burnout, job satisfaction, and overall health, several models have been applied(Table 3). The relationship between the independent and dependent variables (motivation to work) can be interpreted as follows: when controlling for covariates (age, gender, marital status and income, as well as medical setting, working hours, and type of contract), burnout was related to less motivation to work in all three models (personal: β = -0.015, *p* < 0.001; patient-related: β = -0.010, *p* < 0.001; work-related: β = -0.016, *p* < 0.001). Whereas greater job satisfaction (β = 0.143, *p* < 0.001) and overall health (β = 0.014, *p* < 0.001) were related to higher scores on motivation to work and increased motivation to stay in the profession.

**Table 3:** Linear regression models with attitudes towards retirement as the dependent variable

	β-Coefficient	Confidence interval (95%)	*p*-value	Test statistics
**Model 1**: **Personal Burnout**	-0.015	-0.019 – -0.011	*p* < 0.001	F(9, 490) = 8.60; *p* < 0.001 R^2^ = 0.112
**Model 2**: **Patient-rel. Burnout**	-0.010	-0.015 – -0.005	*p* < 0.001	F(8, 485) = 3.82 *p* < 0.001 R^2^ = 0.067
**Model 3**: **Work-related Burnout**	-0.016	-0.021 – -0.012	*p* < 0.001	F(8, 489) = 8.99 *p* < 0.001 R^2^ = 0.122
**Model 4**: **Job satisfaction**	0.143	0.107–0.179	*p* < 0.001	F(8, 472) = 10.40 *p* < 0.001 R^2^ = 0.140
**Model 5**: **Overall health**	0.014	0.008–0.019	*p* < 0.001	F(8, 492) = 5.97 *p* < 0.001 R^2^ = 0.078

*Note* covariates: age, gender, marital status and income, as well as working time (full-time vs. part-time), medical setting (outpatient vs. inpatient) and working condition (permanent, fixed-term contract or self-employed); CI = confidence interval

## Discussion

The aim of the study was to analyze the relationship between motivation to work (by attitudes towards retirement) and possible influencing factors (job satisfaction, health, and burnout) in a broad sample of physicians.

Interestingly, with regard to **age**, we did not find a significant association with motivation to work or retirement. However, age was related to burnout. Previously, it has been shown that early-career physicians show higher levels of burnout compared to their older colleagues, especially women, who are often overrepresented in this profession [21, 22]. This could, in turn, lower motivation to work. However, results regarding the impact of age on the intention to leave direct patient care are mixed [12]. This may also apply to attitudes towards retirement and the motivation to work. More research is needed to fully understand the association between age, burnout, and motivation to work.

According to our study, significant differences between men and women were only observed in terms of work-related and personal burnout but not regarding motivation to work (attitudes towards retirement). **Gender**-related differences have also been postulated before, as women in general stay longer in their profession compared to men, even if they desire to retire earlier [12, 23]. However, as reviewed by Silver et al. [3] gender does not seem to be a relevant factor regarding physicians‘ attitudes towards retirement. Therefore, more research is needed to investigate this relationship and determine gender-specific reasons for the timing of retirement.

According to the literature, having a partner or being married could decrease the motivation to work [23, 24], leading to early retirement for two reasons: financial security (if the partner is able to co-support the relationship financially) and greater interest in activities outside of work (e.g., spending time with the partner). However, it has been shown that physicians fear changes in their relationships with their partners as a result of retirement, which may influence their motivation to work [25]. In our sample, there was no significant relationship between marital status and motivation to work or attitudes towards retirement. Therefore, other confounding variables, which were not part of the current investigation, may influence this relationship.

According to the results of the current study, income was not related to motivation to work or attitudes towards retirement. Previous research suggests a U-shaped relationship between motivation to work and income, so that employees in the low or high-income group (compared to the middle-income group) have the highest motivation to work longer [15]. Studies investigating physician samples suggest that low salary satisfaction or less salary progression results in a higher intention to leave patient care in general than income per se [12]. Inadequate financial preparation has also been found to be a major concern for physicians and may lead to later retirement [26, 27]. In the current study, the range of income may have been too small compared to other studies, and therefore no significant association could be observed. Besides, the income variable in this study was based on household income and not personal income. The relationship between income and motivation to work or attitudes towards retirement may differ depending on whether wages are seen as a financial reward or financial security, so attitudes towards retirement are a question of “affordability.”

So far, there is a lack of studies demonstrating associations between retirement attitudes by physicians and work-related characteristics (such as working hours, type of contract). The results of the current study indicate that working time (full-time or part-time) may be associated with greater motivation to continue work. This is an interesting finding, especially in the context of burnout and motivation to work. Due to the relationship between working hours, work motivation, and burnout [28], one would expect that physicians working full-time may be more motivated towards retirement (instead of continuing to work) compared to part-time physicians, because full-time may be associated with greater overwork hours or a generally higher workload. On the other hand, physicians working part-time may have more time pressure and are therefore less motivated to continue to work due to higher work-related stress.

However, it may be the case that other work-related factors have more explanatory power, as it has been shown that the number of weekend duties or long working hours has a great impact on the intention to leave patient care [12].

According to analyses in our study, being self-employed and working in outpatient care may be associated with greater motivation to continue to work to some extent. On the one hand, physicians working in their own practice may be more driven to continue working and retire later than usual for several reasons, such as not easily finding a successor, especially in rural areas [29]. On the other hand, being self-employed and/or working in outpatient care may be characterized by occupational characteristics that lead to more motivation in a positive way. However, this question can not be answered by the current data set and may be followed up by future studies.

The association with job satisfaction and occupational health was also investigated in the current study using regression models. In our sample, we find higher motivation to work in physicians reporting greater levels of job satisfaction and overall (subjective) health, whereas burnout was negatively associated with motivation to work and attitudes towards retirement. Previous research has shown that physicians with high levels of (autonomous) work motivation also report better occupational **health** in terms of health status, psychosocial stress, burnout, depression, and job satisfaction [12, 30]. Here, the authors find significant associations between occupational health and higher intent to leave the medical profession. In this context, poorer mental health and high burnout were related to a higher intention to leave. Recently, it has been shown that high levels of work-related, personal and especially patient-related burnout can be associated with a tendency for early retirement in GPs and other medical specialties [4, 12, 31, 32] by decreasing the average retirement age by 4.6 years [33]. Therefore, it has been suggested that the costs of burnout associated with early retirement and reduction of clinical hours may substantially impact the health care system. A study by Dewa and colleagues has estimated the costs of physicians experiencing symptoms of burnout to be $185.2 million due to early retirement and $27.9 million due to reductions in clinical hours [34]. Therefore, interventions that aim to reduce the overall burnout of physicians should be developed and implemented to prevent early retirement and physician shortages in the long term. Even if a physician’s intention to retire early may not always result in early retirement, this intention is closely related to actual behavior [3].

### Limitations

The current data set is based on a baseline survey of a longitudinal study and offers first explorative insights but no insights regarding causalities. Data from follow-up surveys could be used to test for possible causalities and investigate whether attitudes change across the lifespan, for instance, due to changes in (personal) priorities with regard to career or family plans or whether there are differences with regard to different generations [35].

One limitation of this study is that it does not differentiate between medical specialties. As many physicians in this study stated that they have more than one specialty, it was not possible to clearly categorize them. In addition, future studies may want to focus on older physicians when investigating attitudes towards retirement. Younger physicians may think of leaving patient care or changing the profession or their workplace as a result of a high workload or dissatisfaction instead of early retirement. In order to test the generalizability of the results, the study could be replicated by focusing on differences between specialties as well as regions (within and outside of Germany). In addition, effect sizes are modest. This should be considered when interpreting the results.

### Implications

This is the first study that addresses this issue based on a broad sample of physicians working in Germany. Therefore, no comparison to similar studies in German settings can be made. Since working conditions within health care are often the result of national health systems and legal regulations, the findings of our study may not be generalizable to health care systems in other countries. However, physician shortage is a globally growing issue. Therefore, it should be investigated whether already existing system-level interventions established in other countries may be suitable to address causes of early retirement and help to keep physicians within health care systems [36]. In this context, the question arises as to how structural aspects of physician‘s work (e.g., working hour arrangements) can be designed in order to compensate for unavoidable staff shortages and at the same time meet physicians‘ wishes for more flexibility and autonomy. In Europe, there have been many changes in legislation (such as maximum working hours, timekeeping and periods of rest), but also in relation to working hours^2^. Clearly, it is of great relevance to investigate out how healthcare is organized and how healthcare workers can be protected and supported. For instance, it may be interesting to investigate how interventions that aim to improve job satisfaction and/or reduce burnout in physicians may also lead to physicians changing their minds about early retirement [37].

The current study results also reveal that some physicians may work beyond their retirement age. According to previous literature, reasons that contribute to late retirement are a sense of responsibility for patients as well as institutional flexibility (e.g., being able to reduce working hours), and overall job satisfaction [3, 4]. Future studies could further investigate these reasons in terms of motivation to work and how they may change over time.

## Conclusion

In conclusion, satisfaction, health, and all three dimensions of burnout were significantly associated with motivation to work, and hence, attitudes towards retirement. The current study builds on previous findings, indicating that motivation to work and attitudes towards retirement among physicians with different medical specialties are complex and multi-faceted issues. The need to further improve physician’s working conditions is highlighted by the results of the current study. Interventions and strategies should be established, that aim to reduce overwork hours, offer flexible working time arrangements, or focus on stress or burnout prevention, tailored to physicians‘ career stages and specific needs (Silver et al., 2016). Creating a future-oriented working environment in the healthcare sector could help to make the medical profession more attractive in the long term and encourage young doctors to work in patient care. At the same time, early retirement may be prevented, and older physicians can act as a valuable resource of knowledge and experience.

### Electronic supplementary material

Below is the link to the electronic supplementary material.

Supplementary Material 1

## Data Availability

“The data that support the findings of this study are available from the corresponding author, [FJ], upon reasonable request due to ethical and third party restrictions.”

## Footnotes

1 MTW meaning „Motivation to work“ [15].

2 According to the Working Time Directive by the European Union (93/104/EC, 11/23/1993), as well as rulings by the Court of Justice of the EU (e.g. EJC C-151/02, 09/09/2003, ECJ C-14/04, 12/01/2005).
