# Supplementary material for: Motivation to work and attitudes towards retirement among physicians
Source: BMC Health Serv Res. 2024 Jul 25;24:846. doi: 10.1186/s12913-024-11296-2 (PMC11282656; doi:10.1186/s12913-024-11296-2)
Supplement: Supplementary file 1 — Supplementary Material 1 [file 12913_2024_11296_MOESM1_ESM.docx]

1. **Overall health Status**

To help you assess how good or bad your health is, we have drawn a scale on the following page, similar to a thermometer. The best conceivable state of health is marked with a “100”, the worst with “0”.

**0**

**100**

**1**

**0**

**2**

**0**

**3**

**4**

**5**

**6**

**7**

**8**

**9**

**0**

**0**

**0**

**0**

**0**

**0**

**0**

**Best imaginable**

**health status**

We would now like to ask you to mark on this scale how good or bad you think your personal health is today.

**Worst imaginable**

**health status**

References:

EuroQol Group. EuroQol - a new facility for the measurement of health-related quality of life. Health Policy 1990; 16(3):199–208.

Herdman M, Gudex C, Lloyd A, Janssen M, Kind P, Parkin D et al. Development and preliminary testing of the new five-level version of EQ-5D (EQ-5D-5L). Qual Life Res 2011; 20(10):1727–36

1. **Copenhagen Burnout Inventory (CBI)**

Below you will find some statements about feelings that relate to your work. To what extent do these apply to you?

|  | **always** |  |  |  | **Never/**  **almost never** |
| --- | --- | --- | --- | --- | --- |
| How often do you feel tired? | O | O | O | O | O |
| How often are you physically exhausted? | O | O | O | O | O |
| How often are you emotionally exhausted? | O | O | O | O | O |
| How often do you think: ’’I can’t take it anymore’’? | O | O | O | O | O |
| How often do you feel worn out? | O | O | O | O | O |
| How often do you feel weak and susceptible to illness? | O | O | O | O | O |

|  | **to a very high degree** |  |  |  | **to a very low degree** |
| --- | --- | --- | --- | --- | --- |
| Is your work emotionally exhausting? | O | O | O | O | O |
| Do you feel burnt out because of your work? | O | O | O | O | O |
| Does your work frustrate you? | O | O | O | O | O |
| Do you feel worn out at the end of the working day? | O | O | O | O | O |
| Are you exhausted in the morning at the thought of another day at work? | O | O | O | O | O |
| Do you feel that every working hour is tiring for you? | O | O | O | O | O |
| Do you have enough energy for family and friends during leisure time? | O | O | O | O | O |
| Do you find it hard to work with patients? | O | O | O | O | O |
| Do you find it frustrating to work with patients? | O | O | O | O | O |
| Does it drain your energy to work with patients? | O | O | O | O | O |
| Do you feel that you give more than you get back when you work with patients? | O | O | O | O | O |
| Are you tired of working with patients? | O | O | O | O | O |
| Do you sometimes wonder how long you will be able to continue working with patients? | O | O | O | O | O |

References:

Kristensen TS, Borritz M, Villadsen E, Christensen KB. The Copenhagen Burnout Inventory: A new tool for the assessment of burnout. Work & Stress 2005; 19(3):192–207.

Klein J. Psychosoziale Arbeitsbelastungen, Burnout und Versorgungsqualität - Eine bundesweite Befragung von Chirurgen [Kumulative Dissertation]. Hamburg: Universität Hamburg; 2013.

1. **Job Satisfaction**

We would also like to know how satisfied you are with your work. For each statement, please tick the box that you think best applies to you.

|  | **agree** | **disagree** |
| --- | --- | --- |
| I have a really interesting job. | O | O |
| After the work is done, I often have the feeling that I have really achieved something. | O | O |
| I think I enjoy my job more than other people. | O | O |
| I believe that I am more satisfied with my work than others. | O | O |
| If I could, I would like to change careers. | O | O |
| Most of the time I enjoy going to work. | O | O |
| The daily monotony of operations often gets on my nerves. | O | O |
| I am quite satisfied with my current work. | O | O |
| I often have a dislike for my work | O | O |
| I often get bored at work. | O | O |

Reference:

Weyer G, Hodapp V, Neuhäuser S. Subjektive Zufriedenheit und Belastung von Arbeit und Beruf; 1997

1. **Motivation To Work Scale**

To what extent do the following statements apply to you? Please use the answer scale (1-5) to answer the questions.

|  | 1  Does not apply at all | 2 | 3 | 4 | 5  Fully applies |
| --- | --- | --- | --- | --- | --- |
| It is highly likely that I will work up to the legal retirement age | O | O | O | O | O |
| The earlier I can quit working the better. | O | O | O | O | O |
| I cannot even imagine that I am ever going to stop working | O | O | O | O | O |
| I feel I am going to miss my work when I think about  retirement. | O | O | O | O | O |
| I would like to work beyond statutory retirement age’ | O | O | O | O | O |

References:

Kanfer R, Beier ME, Ackerman PL. Goals and motivation related to work in later adulthood: An organizing framework. European Journal of Work and Organizational Psychology 2013; 22(3):253–64;

Hasselhorn HM, Ebener M, Vratzias A. Household income and retirement perspective among older workers in Germany-Findings from the lidA Cohort Study. J Occup Health 2020; 62(1):e12130

Stiller M, Garthe N, Hasselhorn HM. Job quality trajectories among baby-boomers in Germany and their consequences for the motivation to work – results from the lidA cohort study. Ageing and Society. 2023;43(7):1638-1660. doi:10.1017/S0144686X21001343
